# SOX13 is a novel prognostic biomarker and associates with immune infiltration in breast cancer

**DOI:** 10.3389/fimmu.2024.1369892

**Published:** 2024-04-19

**Authors:** Ting Gao, Baohong Jiang, Yu Zhou, Rongfang He, Liming Xie, Yuehua Li

**Affiliations:** ^1^ The First Affiliated Hospital, Hengyang Medical School, University of South China, Hengyang, Hunan, China; ^2^ The First Affiliated Hospital of Guangxi Medical University, Nanning, China

**Keywords:** SOX13, prognosis, breast cancer, immunohistochemistry, bioinformatics analysis

## Abstract

**Background:**

The transcription factor, SOX13 is part of the SOX family. SOX proteins are crucial in the progression of many cancers, and some correlate with carcinogenesis. Nonetheless, the biological and clinical implications of SOX13 in human breast cancer (BC) remain rarely known.

**Methods:**

We evaluated the survival and expression data of SOX13 in BC patients via the UNLCAL, GEPIA, TIMER, and Kaplan-Meier plotter databases. Immunohistochemistry (IHC) was used to verify clinical specimens. The gene alteration rates of SOX13 were acquired on the online web cBioportal. With the aid of the TCGA data, the association between SOX13 mRNA expression and copy number alterations (CNA) and methylation was determined. LinkedOmics was used to identify the genes that co-expressed with SOX13 and the regulators. Immune infiltration and tumor microenvironment evaluations were assessed by ImmuCellAI and TIMER2.0 databases. SOX13 correlated drug resistance analysis was performed using the GDSC2 database.

**Results:**

Higher SOX13 expression was discovered in BC tissues in comparison to normal tissues. Moreover, increased gene mutation and amplification of SOX13 were found in BC. Patients with increased SOX13 expression levels showed worse overall survival (OS). Cox analysis showed that SOX13 independently served as a prognostic indicator for poor survival in BC. Further, the expression of SOX13 was also confirmed to be correlated with tumor microenvironment and diverse infiltration of immune cells. In terms of drug sensitivity analysis, we found higher expression level of SOX13 predicts a high IC50 value for most of 198 drugs which predicts drug resistance.

**Conclusion:**

The present findings demonstrated that high expression of SOX13 negatively relates to prognosis and SOX13 plays an important role in cancer immunity. Therefore, SOX13 may potentially be adopted as a biomarker for predicting BC prognosis and infiltration of immune cells.

## 1 Introduction

Breast cancer (BC) is the most common malignant tumor in women. According to the global cancer data in 2020, there were about 2.3 million new cases of BC and 685000 deaths (1). Its annual morbidity and mortality rank first among female malignant tumors (2). According to its infiltration level, BC can occur as either pre-invasive or invasive carcinoma (3). BC develops in stages in clinical practice, starting with normal ductal epithelium and progressing through hyperplasia, *in situ* ductal carcinoma, invasive malignancy, and ultimately metastatic carcinoma. Patients with early-stage BC have a good prognosis, but 20-30% of them will experience local recurrence or distant metastasis within two years after diagnosis (4). It has been reported that the overall survival (OS) rate of primary BC has reached nearly 90% within 5 years. However, the 5-year OS rate of patients with distant non-lymph node metastasis drops to 23% (5, 6). Despite many reports elucidating molecular mutations associated with carcinogenesis, the potential mechanisms of BC progression remain elusive (7–9). Surgery, radiotherapy, chemotherapy, targeted therapy, endocrine therapy, and immunotherapy are the standard treatment modalities for BC (10, 11). Appropriate treatment regimens need to be selected based on clinical and pathological characteristics such as estrogen receptor (ER), progesterone receptor (PR), human epidermal growth factor receptor 2 (HER-2), TNM stage, tumor grade, etc. (12). Currently, the treatment of BC has certain effects, but for some special molecular subtypes, such as triple negative breast cancer (TNBC), the possibility of developing drug resistance is higher, and the treatment effect is poor (13–17). We still know very little about the potential pathogenesis of BC invasiveness, therefore, there is an urgent need for extensive research on the pathogenesis of BC to discover effective drugs and biomarkers.

SOX13 has been revealed to be an essential regulatory element for cell differentiation and cell stemness in both normal and cancer tissues via the regulation of Wnt/β-catenin signaling (18). The expression of SOX13 is high in numerous solid types of tumor, including hepatocellular carcinoma (19), colorectal cancer (20), and gastric cancer (18). As a tumor biomarker with diagnosis and treatment potential in various tumors, high SOX13 expression is linked to poor prognosis. However, the role of SOX13 in BC is still unclear.

Herein, we assessed the expression of SOX13 and its clinical importance in BC by using Immunohistochemistry (IHC), and bioinformatics analysis web servers, including UALCAN, TIMER2.0, GEPIA, etc.

## 2 Materials and methods

### 2.1 TIMER 2.0

TIMER 2.0 (http://timer.cistrome.org/) is a comprehensive online tool, it is vital to analyze immune infiltrates across various cancer (21). With TIMER, users can input function-specific features, whereby the resulting figures are dynamically highlighted to extensive explore the tumor’s clinical, genomic, and immunological characteristics. In our study, TIMER was applied to explore the tumor differential expression with normal tissues for SOX13 among all The Cancer Genome Atlas (TCGA) tumors. Besides, TIMER adopts a deconvolution technique (22), inferring the tumor-infiltrating immune cell (TIIC) abundance according to gene expression profiles. The association between SOX13 expression and immune cell infiltration levels were analyzed.

### 2.2 UALCAN

UALCAN (http://ualcan.path.uab.edu) is a free online resource, it is vital in elucidating the relative transcriptional expression of potentially interesting genes in both normal and tumor samples and the correlation between the transcriptional expression and relative clinicopathologic features (23). Herein, UALCAN was adopted to assess the relative gene expression across normal and tumor samples. Besides, it was applied to prognosis analysis.

### 2.3 GEPIA

GEPIA database (http://gepia.cancer-pku.cn/), an online web, incorporates over 8000 normal samples and over 9000 tumors from TCGA and Genotype-Tissue Expression (GTEx) (24). Using GEPIA, we generated OS curves relying on the median GENE expression level. The remaining default settings of the operating system were all accepted.

### 2.4 Kaplan-Meier plotter

Kaplan-Meier plotter (http://kmplot.com/analysis/) is capable of assessing how over 50000 genes affect survival by analyzing over 1000 cancer samples in 21 cancer types (25). We investigated the relationship between SOX13 expression and BC survival using the Kaplan-Meier plotter and differential gene expression analysis in tumor and normal tissues.

### 2.5 cBioportal

The data on gene mutation, amplification, and methylation was obtained from the cBioportal database (https://www.cbioportal.org/). The methylation data are from the HM450 types, both the association between the SOX13 expression and the copy number alterations and methylation were calculated the Pearson’s correlation coefficients. In addition, the “mafCompare” function in the “maftools” package was used to compare the mutation levels between high and low SOX13 expression groups.

### 2.6 LinkedOmics database analysis

The LinkedOmics database (http://www.linkedomics.org/login.php), is adopted for exploring 32 TCGA cancer-associated multi-dimensional datasets (26). The co-expression of SOX13 was statistically assessed by Pearson’s correlation coefficient, whereby heatmaps or volcano plots were generated.

### 2.7 ImmuCellAI

The ImmuCellAI database was utilized for downloading data of immune cell infiltration of TCGA (http://bioinfo.life.hust.edu.cn/ImmuCellAI#!/). We analyzed the association between SOX13 expression and immune cell infiltration, MHC genes, immunosuppressive genes, chemokine, and chemokine receptors in pan-cancer level. The visualization of all heatmaps in this step was conducted by the “ggplot2” package.

### 2.8 GDSC2

Drug resistance analysis was performed using data from the GDSC2 database (https://www.cancerrxgene.org/). The spearman-correlation analysis was employed to investigate the correlation between drugs and SOX13 expression level, and the drugs with the top 3 strongest positive and negative correlations were displayed respectively. The IC50 differences of the six drugs in high and low SOX13 expression groups were plotted.

### 2.9 Patients

Between January 2013 and December 2019, we retrospectively gathered data on 167 BC patients from the Medical Oncology Department at The First Affiliated Hospital of the University of South China. The following were the eligibility requirements (1): All patients had to have BC that was pathologically proven (2); patients were not given any anticancer medication prior to diagnosis (3); paraffin-embedded tissue samples were available for immunohistochemistry analysis (4); There were complete and searchable medical records. The following criteria were excluded from this study (1): complicated with other malignant tumors or distant metastases (2); lost to follow-up. To learn more about the patient’s outcomes, a follow-up by phone or medical record was conducted. The primary endpoints of this trial were OS, and the follow-up period ended on July 4, 2022. This study was exempted from the requirement of informed consent and approved by the Ethics Committee of The First Affiliated Hospital of University of South China (N0.2021ll0104001).

### 2.10 IHC

The paraffin-embedded tissue samples of 167 BC patients were made a tissue chip wax block (**Supplementary Figure 1**). IHC was carried out in accordance with industry standards. Briefly, the tissue sections were boiled for 15 minutes for antigen repair in citric acid (PH6.0) antigen retrieval solution following deparaffinization and hydration. Endogenous peroxidase activity was inhibited by treatment with a 3 percent methanol solution. The anti-SOX13 antibody (TA321719S, ORIGENE, USA) was incubated with the samples for a whole night at 4°C. The samples were washed and then exposed to the secondary antibody for 50 minutes. Hematoxylin counterstaining and sealing were done after diaminobenzidine coloration. Light microscopy observations were made of the sections.

The expression of SOX13 was assessed independently by two skilled pathologists who were blinded to the clinical information. Staining intensity and extent were graded using the widely used German semi-quantitative scoring system in several locations (27). Scores were given to each specimen based on the degree of nucleic, cytoplasmic, and membrane staining (0 = no staining, 1 = weak staining, 2 = moderate staining, and 3 = strong staining) and the percentage of stained cells (0 = 0-5 percent, 1 = 5-25 percent, 2 = 26-50 percent, 3 = 51-75 percent, and 4 = 76-100 percent). The final result was determined by multiplying these two scores. Scores of 0–7 were placed in the low expression group, and those of 8–12 were placed in the high expression group.

### 2.11 Statistical analysis

First, categorical variables were compared by the χ2 test. Second, the χ2 test was used to investigate the relationships between SOX13 expression and the clinicopathological parameters of the patients. Third, the bivariate correlations between the study variables were displayed using Spearman’s rank correlation coefficient. Then, OS curves were plotted using the Kaplan-Meier curve, and log-rank tests were employed to compare the differences. At last, for both univariate and multivariate analyses of prognostic variables, Cox regression was used. Differences at P<0.05 were deemed significant in all statistical analyses, which were conducted using SPSS 26.0 and R 4.3.1.

## 3 Results

### 3.1 Elevated expression of SOX13 in BC

**Figure 1:**
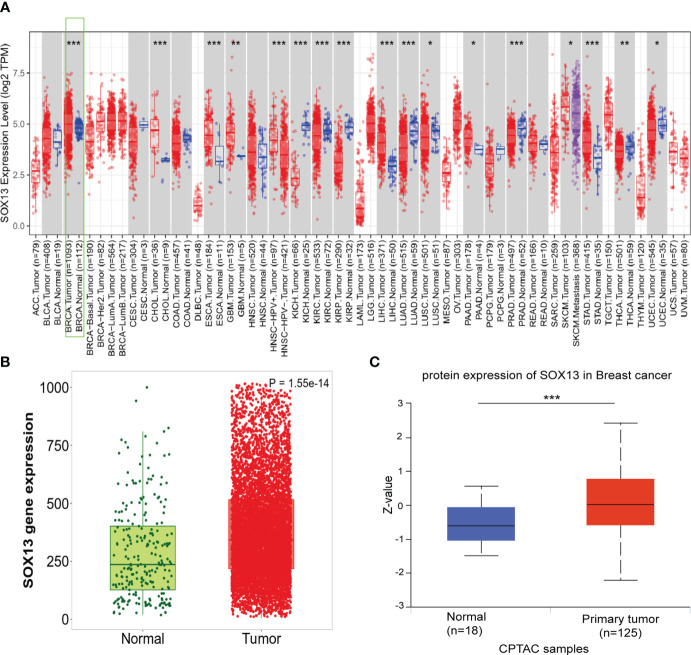
The expression of SOX13 in BC. **(A)**Human SOX13 expression levels were explored by TIMER database in 33 types of cancers (*P < 0.05, **P < 0.01, ***P < 0.001). **(B)** SOX13 expression profiles of normal and tumor tissues by Kaplan–Meier Plotter. **(C)** The protein expression of SOX13 was detected to be overexpressed in BC tissues versus normal samples.

To assess the SOX13 expression in human cancers, we verified the different expression between the adjacent normal tissues and tumor for SOX13 across all TCGA tumors by TIMER database. As was depicted in **Figure 1A**, SOX13 was expressed remarkably higher in cholangiocarcinoma (CHOL), breast invasive carcinoma (BRCA), esophageal carcinoma (ESCA), etc. compared to normal tissues. Notably, SOX13 exhibited markedly lower expression in kidney renal clear cell carcinoma (KIRC), kidney chromophobe (KICH), kidney renal papillary cell carcinoma (KIRP), etc. in comparison to normal tissues. The findings showed that BC had considerably elevated SOX13 expression (p = 0.001) (**Figure 1B**). Moreover, the higher expression of SOX13 protein was reported in BC tissues via the UALCAN database (**Figure 1C**). The protein levels were presented as Z-value which represents standard deviations from the median across samples for breast cancer type. Collectively, these findings demonstrated the high transcriptional and proteinic expressions of SOX13 in BC.

**Figure 2:**
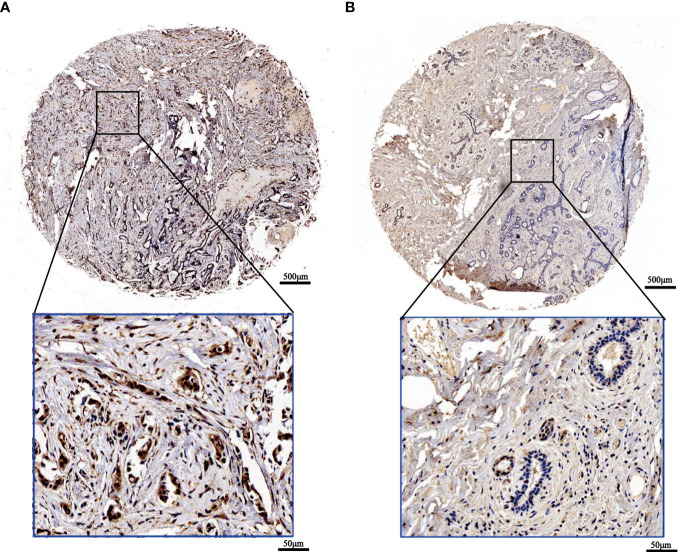
The expression of SOX13 in BC (IHC). **(A)** Tumor **(B)** Normal (upper: bar = 500μm, lower: bar = 50μm).

To confirm the outcomes of the database analysis mentioned above, we performed IHC to test SOX13 protein expression in tissue chip wax block which includes 167 BCs and their counterparts. The results show that the SOX13 proteinic mainly concentrates in the cell nucleus. SOX13 was highly expressed in BC tissues (**Figure 2A**) and lowly expressed in paracancerous tissues (**Figure 2B**).

### 3.2 Gene mutation analysis and methylation analysis

**Figure 3:**
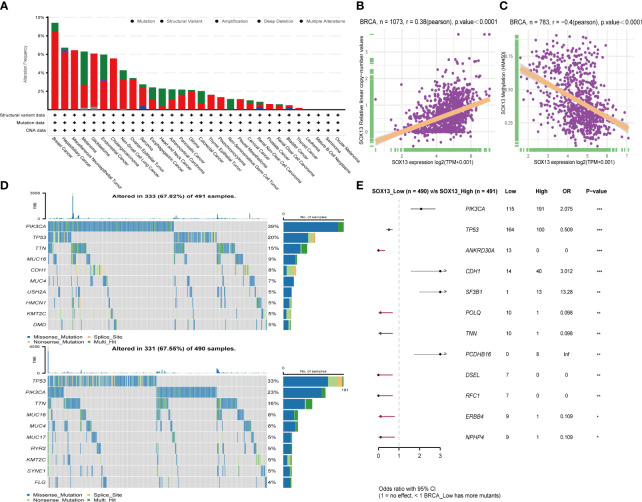
Effect of SOX13 on gene mutations in BC. **(A)** alteration frequency of SOX13 in each cancer of TCGA. **(B)** Correlation between CAN and SOX13 expression. **(C)** Correlation between methylation and SOX13 mRNA expression. **(D)** Top 10 mutated genes in SOX13 high and low expression group. **(E)** Significant different mutation genes in SOX13 low and high expression groups.

First, analysis in the cBioportal database showed the alteration frequency of SOX13 in Pan-cancer. The results showed that the alteration frequency of SOX13 in BC was the highest, and the main type is the amplification mutation (**Figure 3A**). Subsequently, we explored the association between SOX13 and copy number mutation (CNA) in BC. The results showed that SOX13 had a strong positive correlation with BC (**Figure 3B**). Then, we explored the correlation between SOX13 and methylation in BC. The findings demonstrated that SOX13 exhibited a strong negative correlation with BC (**Figure 3C**). In addition, we analyzed the mutation relationship with SOX13 expression. The results showed that the mutation rate of the top 10 mutated genes in the SOX13 high and low expression groups was 67.82% and 67.55%, respectively (**Figure 3D**). We further compared differences in somatic mutations between the two groups. PIK3CA, CDH1, SF3B1, and PCDHB16 had more mutations in SOX13-high group, and genes like TP53 and ANkRD30A had more mutations in SOX13-low group (**Figure 3E**). 

### 3.3 Patient characteristics

**Table 1** lists the characteristics of the patients. The median follow-up period lasted 69 months (with a range of 12 to 113). The range of ages was 29 to 83, with 50 being the median. Patients could be staged as follows: 4 in stage Tis, 43 in stage I, 91 in stage II, 29 in stage III, and 0 in stage IV. Patients’ molecular classification as follows: 36 in Luminal A, 70 in Luminal B, 29 in HER2-E, and 32 in TNBC. 98 patients were grouped in SOX13 low expression, and 69 in SOX13 high expression.

**Table 1:** Patients’ characteristics.

Characteristics	N (%)
Age(y)	
≥50	90(53.9)
<50	77(46.1)
T stage	
Tis	4(2.4)
T1	67(40.1)
T2	85(50.9)
T3	10(6.0)
T4	1(0.6)
N stage	
N0	101(60.5)
N1	40(24.0)
N2	15(9.0)
N3	11(6.5)
TNM stage	
0	4(2.4)
I	43(25.7)
II	91(54.5)
III	29(17.4)
IV	0(0.0)
ER status	
Negative	56(33.5)
Positive	111(66.5)
PR status	
Negative	64(38.3)
Positive	103(61.7)
HER-2 status	
Negative	134(80.2)
Positive	33(19.8)
Molecular classification	
Luminal A	36(21.6)
Luminal B	70(41.9)
HER2-E	29(17.3)
TNBC	32(19.2)
SOX13	
Low	98(58.3)
High	69(41.1)

### 3.4 Association with SOX13 expression and clinicopathologic variables

To confirm the significance of SOX13 expression, IHC staining was performed for a cohort containing 167 cases of BC paired with paracancerous tissue. Results uncovered that SOX13 expression was remarkably related to N stage (p = 0.046) and molecular classification (p = 0.016) (**Table 2**). The expression of SOX13 was substantially connected with molecular categorization (p = 0.012) and vital state (p = 0.022), according to a Spearman analysis of the relationship between SOX13 and clinicopathological parameters (**Table 3**).

**Table 2:** Association between SOX13 expression and clinicopathologic features.

Characteristics	N	SOX13 expression, n (%)		P
		Low	High	
**Age(y)**				0.797
≥50	90	52	38	
<50	77	46	31	
**T stage**				0.421
Tis	4	1	3	
T1	67	43	24	
T2	85	48	37	
T3	10	5	5	
T4	1	1	0	
**N stage**				0.046
N0	101	62	39	
N1	40	25	15	
N2	15	9	6	
N3	11	2	9	
**TNM stage**				0.082
0	4	1	3	
I	43	27	16	
II	91	58	33	
III	29	12	17	
IV	0	0	69	
**ER status**				0.051
Negative	56	27	29	
Positive	111	71	40	
PR status				0.141
Negative	64	33	31	
Positive	103	65	38	
**HER-2 status**				0.802
Negative	134	78	56	
Positive	33	20	13	
**Molecular classification**				0.016
Luminal A	36	25	11	
Luminal B	70	43	27	
HER2-E	29	19	10	
TNBC	32	11	21	

**Table 3:** Spearman analysis of correlation between SOX13 and clinicopathological.

Variables	SOX13 expression level	
	Spearman Correlation	P
T stage	0.042	0.591
N stage	0.106	0.173
TNM stage	0.083	0.286
ER status	-0.151	0.051
PR status	-0.114	0.142
HER-2 status	-0.019	0.804
Molecular classification	0.194	0.012
Vital states	0.177	0.022

### 3.5 Elevated SOX13 expression is correlated with poor outcome in BC patients

**Figure 4:**
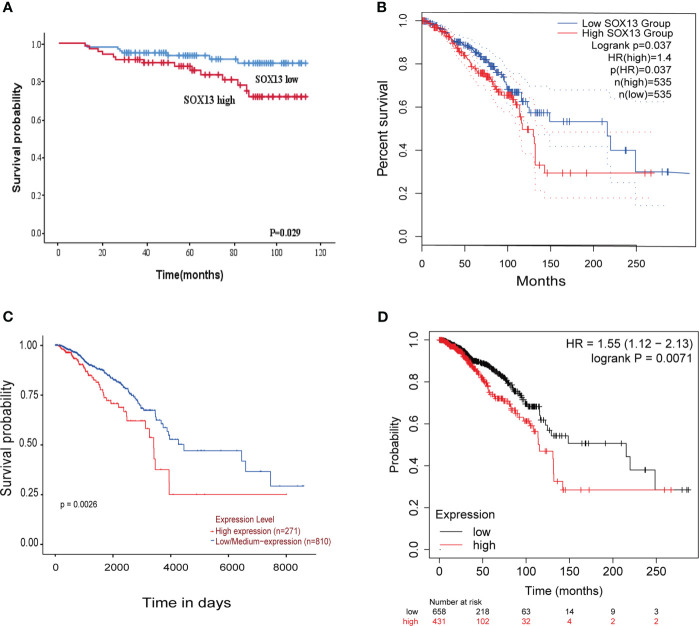
The higher the SOX13 expression, the worse the OS. **(A)** Kaplan-Meier OS curves of BC patients according to the expression of SOX13. **(B)** GEPIA. **(C)** UALCAN. **(D)** Kaplan-Meier plotter.

Here, we analyzed the prognostic potential of SOX13. The Kaplan–Meier survival curves revealed that higher SOX13 levels were associated with worse OS (**Figure 4A**). Furthermore, GEPIA, UALCAN, and Kaplan–Meier plotter databases showed that increased expression of SOX13 correlates with poor outcomes in BC (**Figures 4B–D**).

We also looked at the relative hazards that SOX13 indicated for the prognosis of BC. To assess whether SOX13 might be a risk factor, Cox regression analysis was utilized. According to **Table 4**, T stage (p = 0.009), N stage (p = 0.007), TNM stage (p = 0.002), and SOX13 (p = 0.035) were all linked with OS in univariate analysis. Multivariate Cox regression analysis revealed that T stage and SOX13 were independent prognostic factors. These findings show a substantial relationship between SOX13 expression and BC prognosis.

**Table 4:** Univariate and multivariate Cox proportional hazards analysis of SOX13 expression and OS for BC patients.

Characteristics	Univariate analysis			Multivariate analysis		
	HR	95%CI	P	HR	95%CI	P
Age	1.158	0.500-2.684	0.723			
T stage	2.419	1.250-4.681	0.009	2.438	1.213-4.902	0.012
N stage	1.669	1.148-2.426	0.007	1.163	0.568-2.384	0.679
TNM stage	2.284	1.467-5.434	0.002	2.267	0.567-9.061	0.247
ER status	0.759	0.324-1.777	0.525			
PR status	0.593	0.257-1.368	0.220			
HER-2 status	0.531	0.157-1.798	0.309			
Molecular classification	1.428	0.949-2.148	0.087			
SOX13	2.547	1.068-6.074	0.035	2.457	1.030-5.860	0.043

### 3.6 Co-expression analysis of SOX13

**Figure 5:**
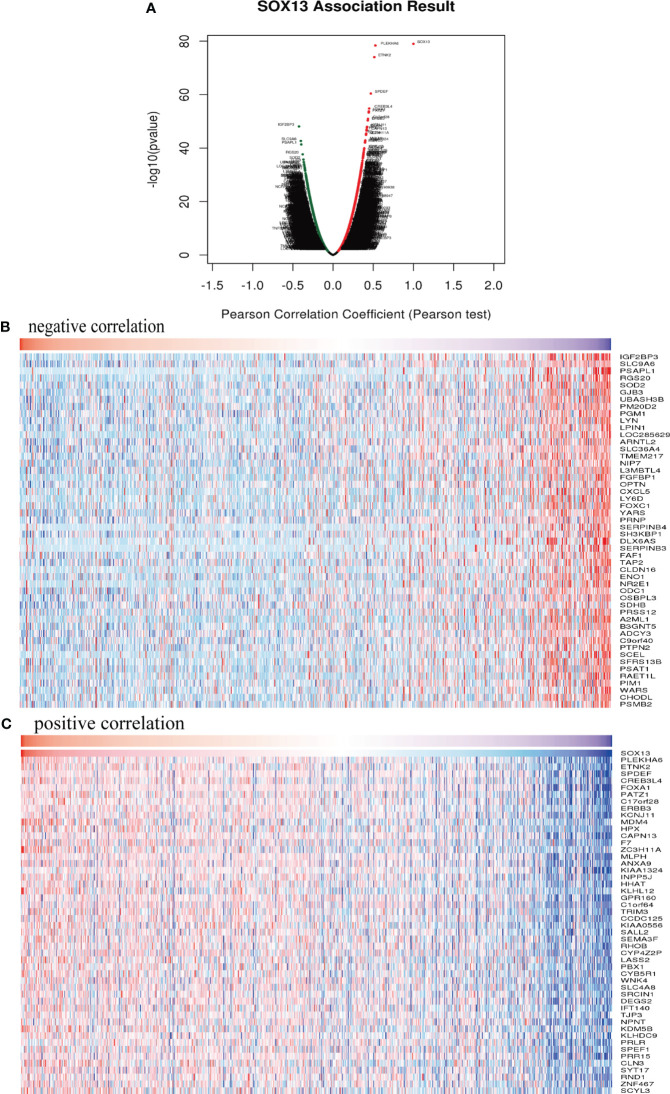
SOX13 co-expression genes in BC (LinkedOmics) and enrichment analysis. **(A)** Genes whose expression was highly associated with SOX13 confirmed by Pearson test in BC cohort. Heatmaps showing the top 50 genes negatively **(B)** and positively **(C)** correlated with SOX13 in BC.

To clarify SOX13’s biological functions in BC, we employed the LinkedOmics function module to evaluate the SOX13 mode of co-expression in the BC cohort. From **Figure 5A**, 4631 genes (dots in dark red) depicted a significant positive association with SOX13. On the other hand, 5316 genes (dark green dots) displayed a markedly negative correlation (FDR< 0.01). The upmost 50 remarkable genes exhibited both negative and positive correlations with SOX13 and have been highlighted in the heat map (**Figures 5B, C**).

### 3.7 Enrichment analysis of co-expression genes correlated with SOX13 in BC

**Figure 6:**
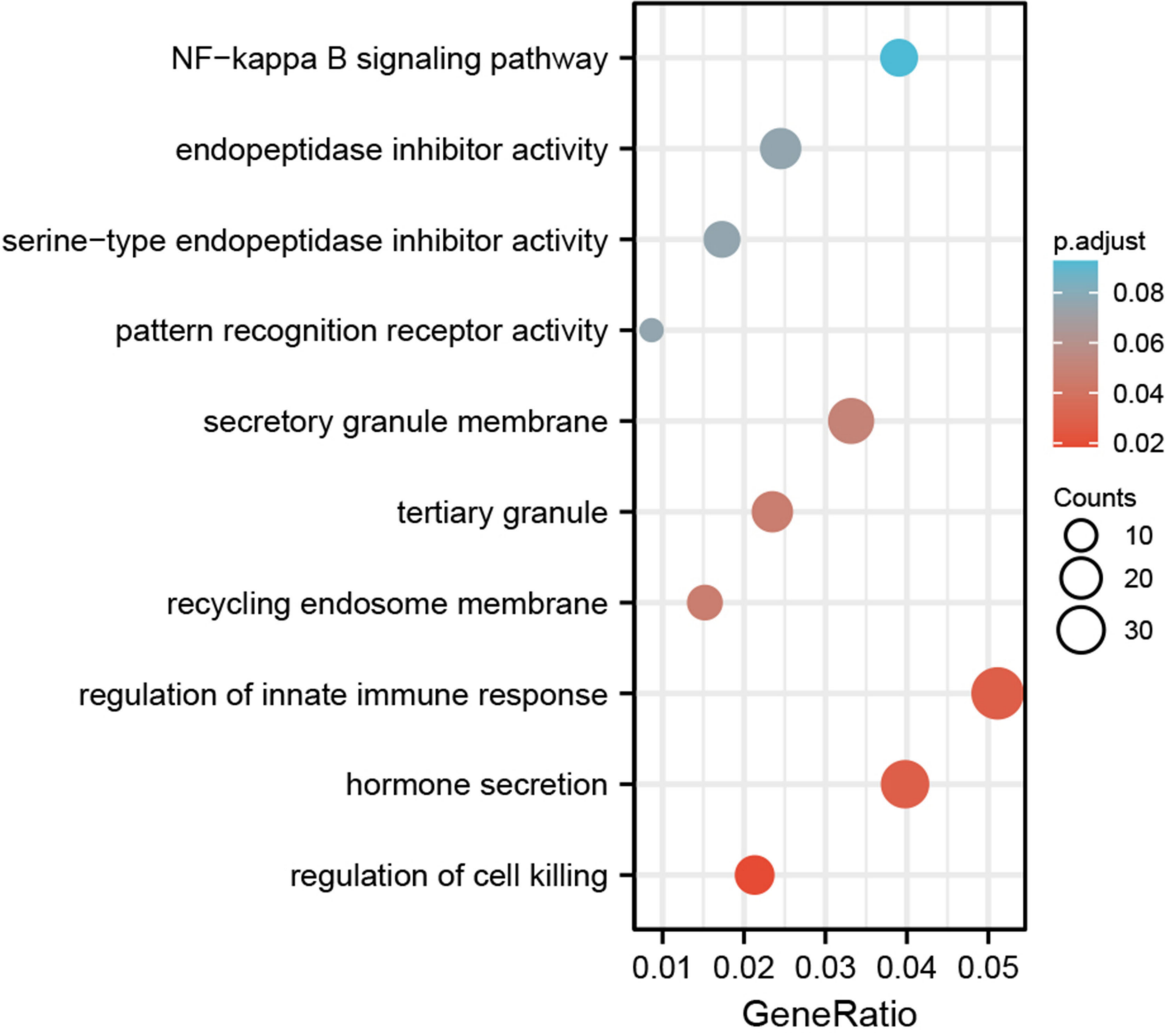
The GO and KEGG enrichment analysis of SOX13 co-expressed genes.

Based on the co-expression gene analysis results of SOX13 (person correlation >0.25, p <0.05), significant GO term analysis through clusterprofiler package in R 3.6.1 highlighted differentially expressed gene, showing correlations with SOX13 were primarily located in the side of secretory granule membrane, recycling endosome membrane and tertiary granule, where they participated primarily in hormone secretion, regulation of innate immune response and regulation of cell killing. Their molecular Function as follows: endopeptidase inhibitor activity, pattern recognition receptor activity, and serine−type endopeptidase inhibitor activity. Enrichment was found in the NF-kappa B signaling pathway by KEGG pathway analysis (**Figure 6**).

### 3.8 The effects of SOX13 on immune cell infiltration

**Figure 7:**
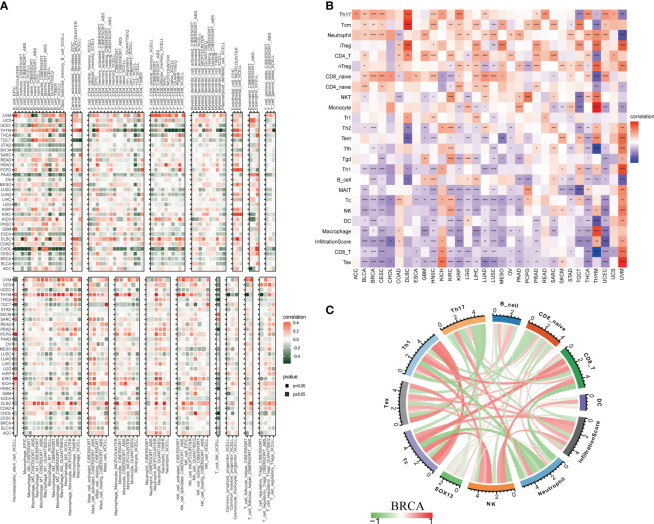
The role of SOX13 in tumor microenvironment. **(A)** The role of SOX13 on immune infiltration using the data from the TIMER2 database. **(B)** The role of SOX13 on immune cell infiltration using data from ImmuCellAI database. **(C)** The role of SOX13 on immune cell infiltration in the setting of breast cancer. *p<0.05, **p<0.01, ***p<0.001, ****p<0.0001.

The role of SOX13 on immune infiltration was figured using the data from the TIMER2 dataset (**Figure 7A**). The results indicated that SOX13 was positively correlated with cancer-associated fibroblasts, endothelial, M2 macrophage, etc., and negatively correlated with CD8+ T cells, myeloid dendritic cells, monocytes, etc. in BRCA. By exploring the correlation of SOX13 expression with immune cell infiltration utilizing the ImmuCellAI database, it was noticed that SOX13 was positively associated with Th17, T cm, neutrophil, and CD8_naive while negatively correlated with Th2, Tfh, Th1, B cell, Tc, NK, DC, macrophage, infiltration score, CD 8+ T, and Tex in BRCA (**Figures 7B, C**).

### 3.9 The association between SOX13 and immune-related genes

**Figure 8:**
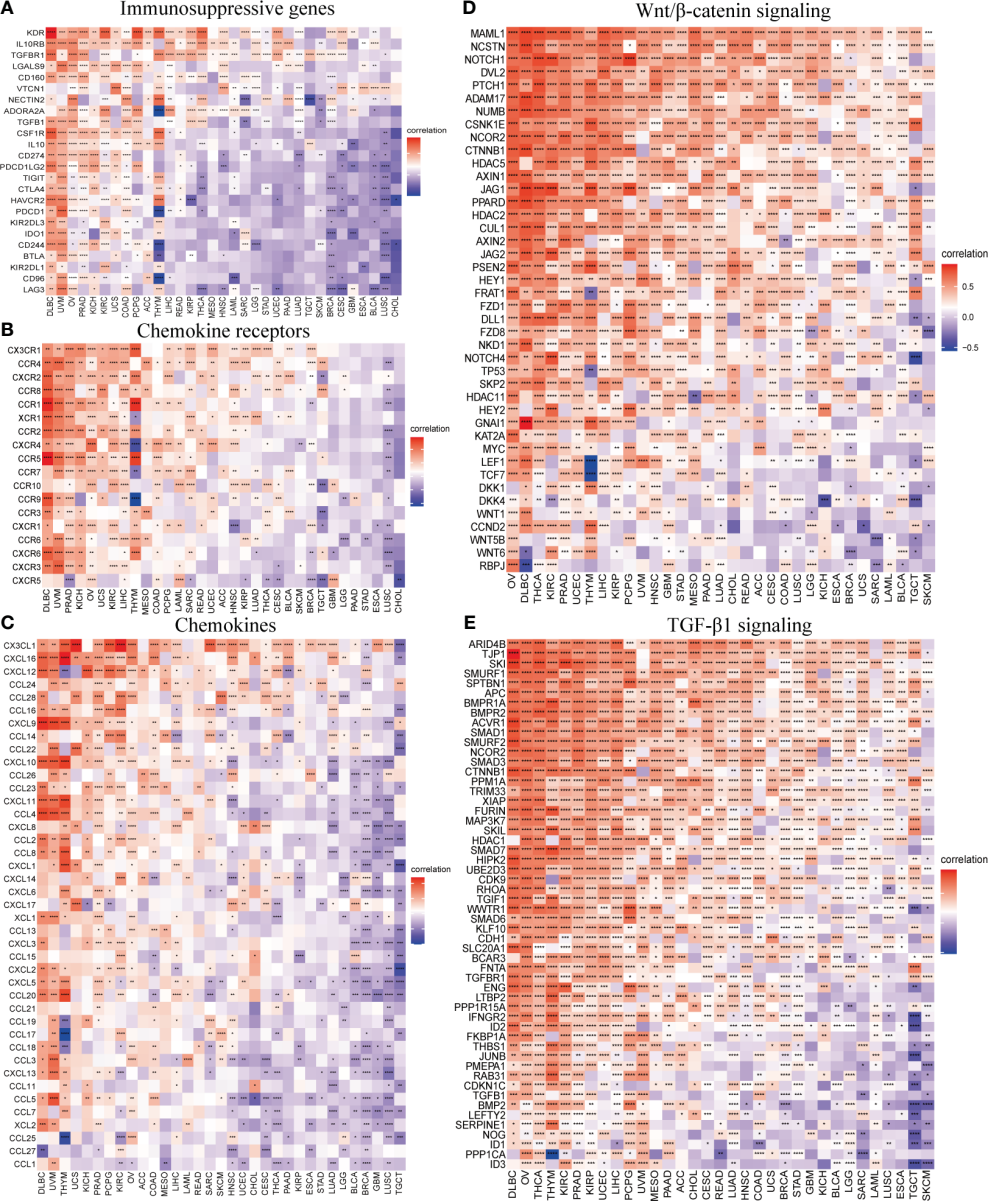
SOX13 correlation with immunomodulatory genes. **(A)** Immunosuppressive genes. **(B)** Chemokine receptor genes. **(C)** Chemokine genes. **(D)** Wnt/β-catenin signaling genes. **(E)** TGF-β1 signaling genes. *p<0.05, **p<0.01, ***p<0.001, ****p<0.0001.

The correlation of SOX13 with expression and immune regulatory genes was further analyzed. The results showed that the SOX13 gene has a potential immunomodulatory effect in most tumors (**Figure 8**). On immunosuppressive genes in BC, SOX13 was positively correlated with KDR, TGFBR1, and NECTIN2 (**Figure 8A**). Chemokine receptors such as CX3CR1, CCR4, and CXCR1 and chemokines such as CXCL12, CCL16, and CCL14 were positively correlated with SOX13 expression in BC (**Figures 8B, C**). Moreover, the association between SOX13 and immune inhibitory genes in BC in specific Wnt/β-catenin and TGF-β1 signaling pathways was also illustrated. In Wnt/β-catenin pathways, the top 3 SOX13 positively correlated genes were MAML1, NCSTN, and NOTCH1, whereas the top 3 negatively correlated genes were RBPJ, WNT6, and WNT5B (**Figure 8D**). In TGF-β1 pathways, the top 3 SOX13 positively correlated genes were ARID4B, TJP1, and SKI, whereas the top 3 negatively correlated genes were ID3, PPP1CA, and ID1 (**Figure 8E**).

### 3.10 SOX13 correlated drug resistance analysis

**Figure 9:**
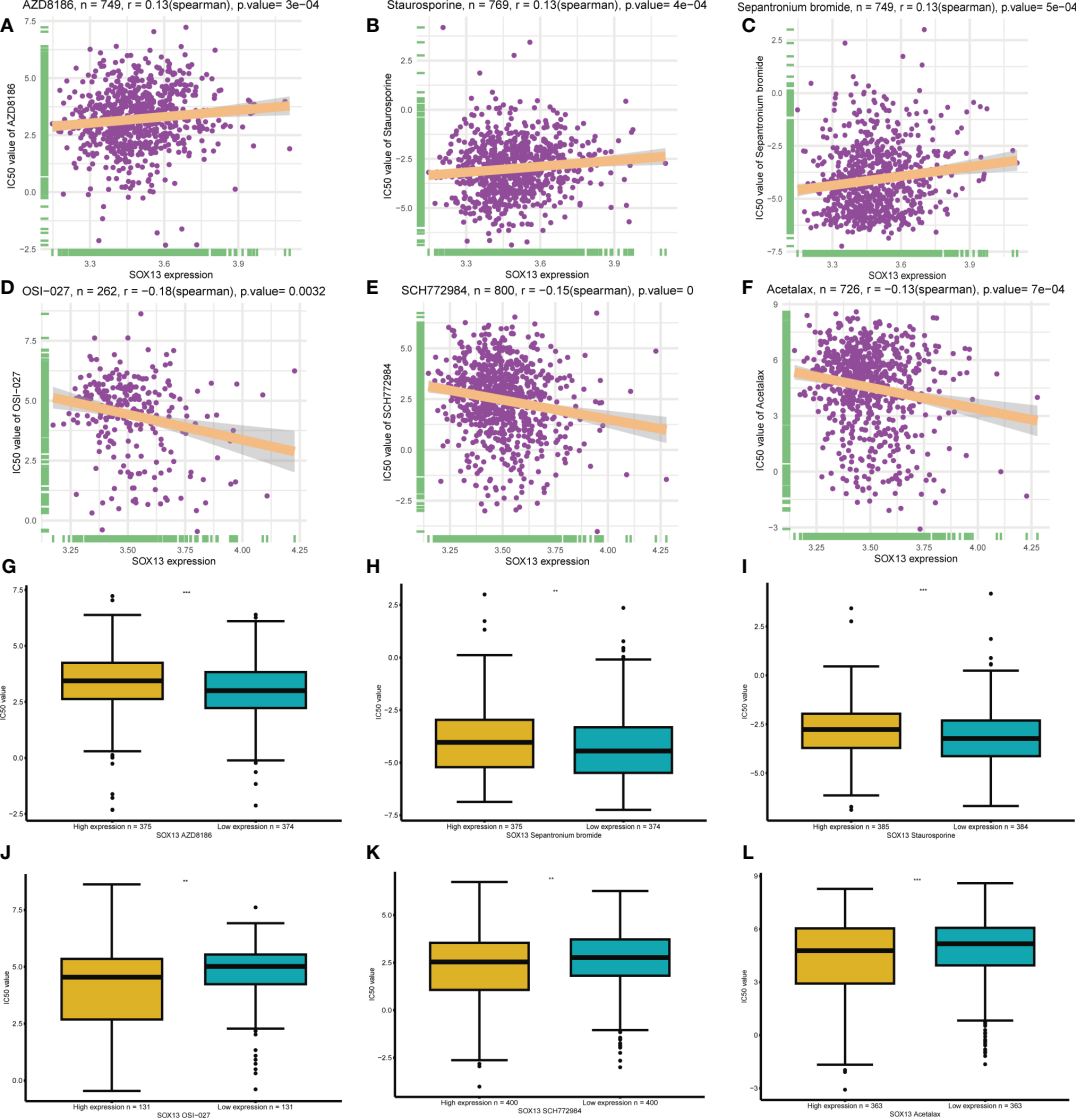
| Drug sensitivity analysis. **(A-C)** The top 3 SOX13 positively correlated drugs from the GDSC2 database. **(D, F)** The top 3 SOX13 negatively correlated drugs from the GDSC2 database. **(G-L)** Differential expression of IC50 between different drugs in high and low SOX13 expression groups.

A total of 198 drugs were identified as being associated with SOX13. The top 3 SOX13 positively correlated drugs from the GDSC2 database were AZD8186, Staurosporine, and Sepantronium bromide (**Figures 9A–C**), and the IC50 of three drugs in the high-risk group was higher than low-risk group (**Figures 9G–I**). In addition, the top 3 SOX13 negatively correlated drugs were OSI-027, SCH772984, and Acetalax (**Figures 9D–F**), and the IC50 of three drugs in the high-risk group was lower than low-risk group (**Figures 9J–L**).

## 4 Discussion

BC is regarded as a highly complex and heterogeneous malignancy. Despite the advancement in diverse therapeutic methods, the OS rate of patients with BC at advanced stages remains elusive (28, 29). Thus, it is imperative that uncover novel factors for predicting and effectively managing BC and devise additional novel treatment measure (30, 31). SOX13, located within the human chromosome 1q31.3–32.1, and a study revealed SOX13 as a specific immune system gene (32). In this study, which described SOX13’s role in BC, it shows that high SOX13 expression is associated with a bad prognosis.

In order to obtain a reliable result, the expression of SOX13 was explored by the TCGA database, and then we confirmed the result with IHC and databases. The present results demonstrated over-expressions of protein and mRNA in SOX13. Therefore, monitoring the expression level of SOX13 may be an effective diagnostic method for BC. Subsequently, the mutation analysis found that the alteration frequency of SOX13 in BC was the highest. We also found that SOX13 expression is influenced by gene mutations, CNA, and DNA methylation. This enriched our understanding of the functionality of SOX13. Expression of SOX13 mRNA was dramatically correlated with nodal stage and molecular classification. Survival analysis showed that SOX13 was an independent prognostic indicator for many tumors. Patients highly expressing SOX13 showed worse OS. Analysis of the immune microenvironment revealed the role of SOX13 in the immune environment including immune-related genes and immune cell infiltration. The final drug sensitivity analysis provided the strongest association with SOX13. This provides an idea for targeting SOX13 therapy.

The association of SOX13 expression with the level of immune infiltration in BC is also a major aspect presented in the present work. Immune checkpoint inhibitors (ICIS), such as PD-1/PD-L1 antibodies, have changed the way many cancers are treated (33).Studies have shown that cells of the immune system are rich in the tumor microenvironment in BC regardless of subtypes, higher rates of pathological complete response were reflected in neoadjuvant chemotherapy (34–36). According to the findings of the present research, SOX13 serves a prominent function in the immune milieu of BC cells. SHEN et al. (37)have shown that M2 macrophages are mainly concentrated in lymph node metastasis positive samples of BC, and M2 macrophages have strong ability of migration and activation *in situ* tumor tissues. We analyzed the correlation between SOX13 and the infiltration of immune cells in BC. The results revealed that SOX13 was positively correlated with cancer-associated fibroblasts, endothelial, M2 macrophage, etc. Besides, the infiltration levels of immune killer cells, including B cells, CD8+ T cells, NK cells, and macrophages were negatively correlated with SOX13 expression in BC. To further explore the connection between SOX13 and the immunosuppressive milieu, we conducted a correlation analysis between immune-related genes and SOX13. Our study found significant correlations of SOX13 with most immunosuppressive checkpoints, including KDR, TGFBR1, and NECTIN2. Meanwhile, our results show that SOX13 also have markable relation with chemokines and chemokine receptors. Wnt/β-catenin and TGF-β1 signaling pathways play an important role in the progression of BC (38, 39). Our study found that SOX13 is significantly associated with genes of Wnt/β-catenin and TGF-β1 signaling pathways. The above results indicate that SOX13 has significance for further exploration in immune regulation in the tumor microenvironment.

In the drug sensitivity analysis, we found higher SOX13 expression in BC indicates a higher IC50 value for most drugs in the GDSC2 database, which shows the measurement of SOX13 expression level may act as a reliable indicator for clinical therapy. Combining with the SOX13 expression and its drug sensitivity analysis, we may establish risk stratification for cancer patients, which optimizes the development and application of anti-cancer drugs. These findings suggest that SOX13 may be a potential target for cancer therapy, which contributes to studying the mechanisms of anti-cancer drug resistance.

Our research has some limitations. The sample size of this study is relatively small, and a larger sample size is needed to verify the results. We need more experiments to verify the expression of SOX13 in BC. In addition, its molecular mechanism was not discussed in this study, and experiments should be carried out *in vivo* and *in vitro*.

## 5 Conclusions

According to the current study, BC has high SOX13 expression. SOX13 is a promising clinical biomarker that can be applied to early diagnosis. In addition, SOX13 was revealed to be remarkably related to unfavorable OS in BC patients, which provides an in-depth understanding of molecular targets for future therapeutic strategies of BC. The above findings potentially guide the establishment of novel strategies for SOX13 in BC.

## Data availability statement

The original contributions presented in the study are included in the article/**Supplementary Material**. Further inquiries can be directed to the corresponding authors.

## Ethics statement

The studies involving humans were approved by The Ethics Committee of The First Affiliated Hospital of University of South China. The studies were conducted in accordance with the local legislation and institutional requirements. This is a retrospective study that exempting informed consent does not have adverse effects on the health and rights of participants, and the privacy and personal identity information of participants are protected.

## Author contributions

TG: Writing – original draft. BJ: Writing – original draft. YZ: Writing – original draft. RH: Writing – original draft. LX: Writing – review & editing. YL: Writing – review & editing.

## Supplementary material

Supplementary Figure 1 Tissue chip and tissue microarray section **(A)** Tissue chip. **(B)** Tissue microarray section
