# Supplementary figures and images for: SOX13 is a novel prognostic biomarker and associates with immune infiltration in breast cancer
Source: Front Immunol. 2024 Apr 19;15:1369892. doi: 10.3389/fimmu.2024.1369892 (PMC11066178; doi:10.3389/fimmu.2024.1369892)

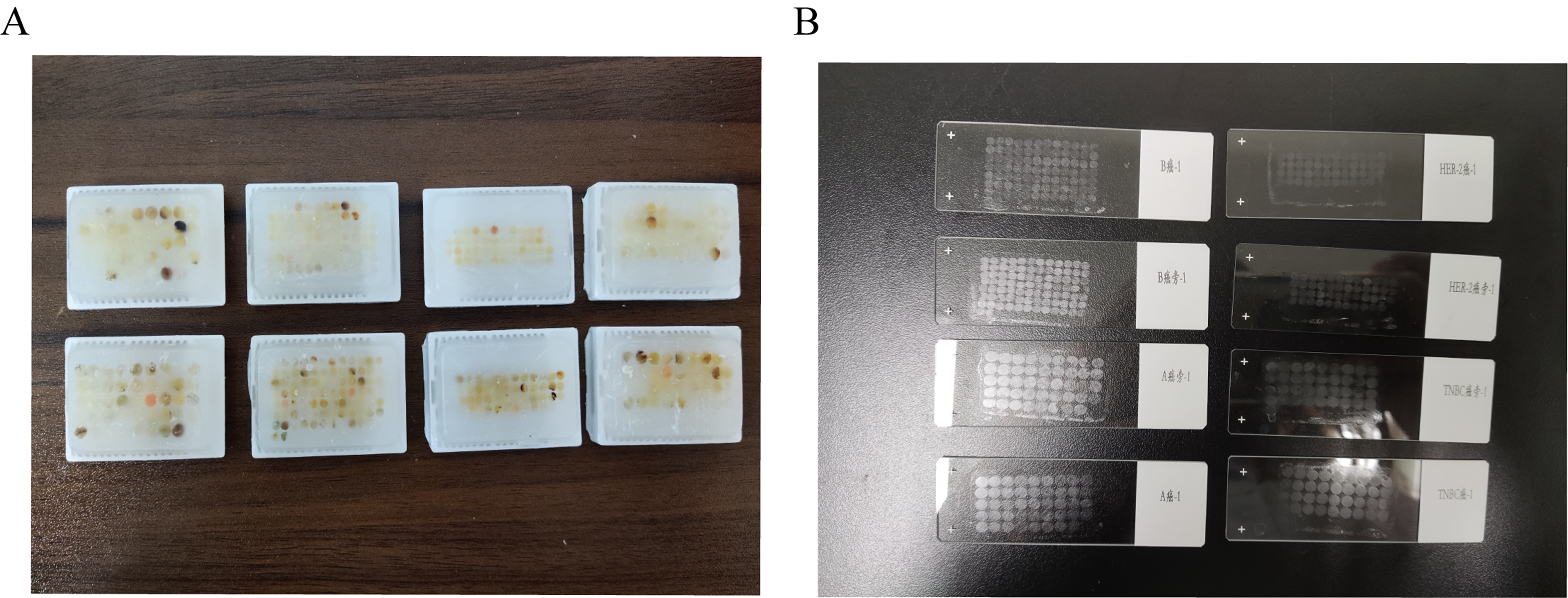

Supplement: Supplementary Figure 1 — Tissue chip and tissue microarray section (A) Tissue chip. (B) Tissue microarray section [file Image_1.tif]
