# Supplementary material for: SOX13 is a novel prognostic biomarker and associates with immune infiltration in breast cancer
Source: Front Immunol. 2024 Apr 19;15:1369892. doi: 10.3389/fimmu.2024.1369892 (PMC11066178; doi:10.3389/fimmu.2024.1369892)
Supplement: Supplementary file 2 [file Table_1.docx]

Raw data link: https://www.jianguoyun.com/p/DSqIV1IQmaWpDBi-pLAFIAA
